# *Staphylococcus aureus* Esx Factors Control Human Dendritic Cell Functions Conditioning Th1/Th17 Response

**DOI:** 10.3389/fcimb.2017.00330

**Published:** 2017-07-21

**Authors:** Melania Cruciani, Marilena P. Etna, Romina Camilli, Elena Giacomini, Zulema A. Percario, Martina Severa, Silvia Sandini, Fabiana Rizzo, Valentina Brandi, Giuliana Balsamo, Fabio Polticelli, Elisabetta Affabris, Annalisa Pantosti, Fabio Bagnoli, Eliana M. Coccia

**Affiliations:** ^1^Department of Science, University Roma Tre Rome, Italy; ^2^Department of Infectious Diseases, Istituto Superiore di Sanità Rome, Italy; ^3^Research Center, GSK Vaccines Siena, Italy; ^4^National Institute of Nuclear Physics, Roma Tre University Rome, Italy

**Keywords:** *S. aureus*, Ess, dendritic cells, vaccine, cytokine, apoptosis, Th response

## Abstract

The opportunistic pathogen *Staphylococcus aureus* (*S. aureus*) is a major cause of nosocomial- and community-acquired infections. In addition, many antibiotic-resistant strains are emerging worldwide, thus, there is an urgent unmet need to pinpoint novel therapeutic and prophylactic strategies. In the present study, we characterized the impact of infection with the pandemic methicillin-resistant USA300 *S. aureus* strain on human primary dendritic cells (DC), key initiators and regulators of immune responses. In particular, among staphylococcal virulence factors, the function of EsxA and EsxB, two small acidic dimeric proteins secreted by the type VII-like secretion system Ess (ESAT-6-like secretion system), was investigated in human DC setting. A comparative analysis of bacterial entry, replication rate as well as DC maturation, apoptosis, signaling pathway activation and cytokine production was performed by using wild type (wt) USA300 and three isogenic mutants carrying the deletion of *esxA* (Δ*esxA*), *esxB* (Δ*esxB*), or both genes (Δ*esxAB*). The *S. aureus* mutant lacking only the EsxA protein (Δ*esxA)* stimulated a stronger pro-apoptotic phenotype in infected DC as compared to wt USA300, Δ*esxAB*, and Δ*esxB* strains. When the mutant carrying the *esxB* deletion (Δ*esxB*) was analyzed, a higher production of both regulatory and pro-inflammatory mediators was found in the infected DC with respect to those challenged with the wt counterpart and the other *esx* mutants. In accordance with these data, supernatant derived from Δ*esxB*-infected DC promoted a stronger release of both IFN-γ and IL-17 from CD4^+^ T cells as compared with those conditioned with supernatants derived from wild type USA300-, Δ*esxAB*-, and Δ*esxA*-infected cultures. Although, the interaction of *S. aureus* with human DC is not yet fully understood, our data suggest that both cytokine production and apoptotic process are modulated by Esx factors, thus indicating a possible role of these proteins in the modulation of DC-mediated immunity to *S. aureus*.

## Introduction

*Staphylococcus aureus* (*S. aureus*) is a highly adaptive Gram-positive coccus that can be either a human commensal or a potentially lethal opportunistic pathogen. Approximately one third of the entire human population worldwide is asymptomatically colonized by *S. aureus*, which mainly inhabits epithelial surfaces (Missiakas and Schneewind, 2016). However, under certain circumstances, this bacterium can eventually become a life-threatening pathogen causing infections in skin and soft tissues, sepsis, endocarditis, osteomyelitis, pneumonia, and toxic shock syndrome (David and Daum, 2010; Olaniyi et al., 2016). *S. aureus* is responsible for infections both in the healthcare setting and in the community and, also due to its impressive capacity to develop antibiotic resistance, constitutes a major burden on human society (Monaco et al., 2016). Indeed, the increasing frequency of both methicillin-resistant *S. aureus* strains (MRSA) and strains with decreased susceptibility to vancomycin has complicated disease treatment (Gould et al., 2012), thus the need to develop new therapeutic strategies and vaccines against this dangerous pathogen becomes urgent.

The remarkable ability of *S. aureus* to cause a wide range of infections is related to its extensive armamentarium of virulence factors, which includes secreted proteins, cell wall anchored proteins and cell surface components that allow the bacteria to adhere to cell surface, invade or escape host immune system and cause harmful toxic effects (Gordon and Lowy, 2008; Bien et al., 2011; Pozzi et al., 2015). In addition, several evidences showed that *S. aureus* possesses a specialized secretion system, namely ESAT-6-like secretion system (Ess), similar to the ESX-1 secretion system described in *Mycobacterium tuberculosis* (Sundaramoorthy et al., 2008; Anderson et al., 2013), which contributes to *S. aureus* virulence and pathogenesis (Burts et al., 2005, 2008). Ess is encoded within the *ess* gene cluster, a region highly conserved in the genomes of both community- and hospital-acquired *S. aureus* strains (Korea et al., 2014). Ess consists of structural components, EssA, EssB, EssC, EssD, EssE, and accessory molecules, including the membrane protein EsaA and the cytosolic proteins EsaB and EssI (Anderson et al., 2016; Warne et al., 2016; Ohr et al., 2017). Secreted substrates of the *S. aureus* Ess machinery include EsxA and EsxB, two small acidic dimeric proteins carrying a distinctive WXG motif, and two additional proteins, EsxC and EsxD, which lack the WXG motif (Jager et al., 2016). Moreover, recent evidences showed that Ess may also secrete the structural factor EssD, which acts as a nuclease to cleave DNA and whose activity is inhibited by EssI (Ohr et al., 2017).

Among the Ess secreted substrates, EsxA and EsxB show similarity to ESAT-6 and CFP-10, two well characterized virulence factors encoded by *M. tuberculosis* (Burts et al., 2005), and are reported to be bacterial effectors that are involved in host-pathogen interaction (Groschel et al., 2016). Mutants unable to secrete EsxA and EsxB displayed defects in *S. aureus*-mediated abscess formation and persistence in host tissue (Burts et al., 2008). Moreover, Ess has also been shown to manipulate host immune responses by modifying the production of specific cytokines (Anderson et al., 2016) and to impact nasal colonization and pneumonia, although in a strain-dependent manner (Kneuper et al., 2014). The mechanisms by which EsxA and EsxB exert their pathogenic function remains to be elucidated, although a recent study performed in epithelial cells indicates a role in the modulation of apoptosis and release of intracellular *S. aureus* from the host cell (Korea et al., 2014). In the present study, we further investigated the mechanism of action of EsxA and EsxB using an *in vitro* human dendritic cells (DC)-based infection model. These studies suggested that Esx factors might modulate DC apoptosis and cytokine production and, in turn, T cell expansion, highlighting the key role played by these factors in regulating the host immune response.

## Materials and methods

### DC preparation and infection

Istituto Superiore di Sanità Review Board approved the present research project (CE/13/387). DC were prepared as previously described (Etna et al., 2015). DC were generated by culturing CD14^+^ monocytes (purified from human peripheral blood mononuclear cells of anonymous healthy blood donors) with 50 ng/ml GM-CSF (R&D Systems, Minneapolis, MN, USA) and 200 U/ml IL-4 (Miltenyi, Bergisch Gladbach, Germany) for 5 days at 0.5 × 10^6^ cells/ml in RPMI 1640 (BioWhittaker Europe, Verviers, Belgium) supplemented with 2 mM L-glutamine (BioWhittaker) and 15% Fetal Bovine Serum (FBS) (Lonza, Basel, Switzerland). At day 5, cells were tested for their differentiation status by evaluating CD1a expression (>90% CD1a^+^) and lack of CD14 (>95% CD14^−^). Before infection, the medium was replaced with RPMI without antibiotics and supplemented with 2 mM L-glutamine and 15% FBS. Cytokine deprivation did not affect DC survival rate, which was >90%.

DC were then infected with *S. aureus* cultures grown as described above using a multiplicity of infection of 0.1 bacteria/cell. After 2 h of infection, extracellular bacteria were killed by adding gentamicin (50 μg/ml, Sigma Aldrich) for 2 h at 37°C. Then, DC cultures were washed with growth medium before plating in antibiotic-free medium.

### Bacterial strains and growth conditions

*S. aureus* wt USA300 strain and three isogenic mutants, namely Δ*esxAB*, Δ*esxA*, and Δ*esxB* carrying the deletions in both *esxA* and *esxB*, or only *esxA* or *esxB* genes, respectively, were used to infect DC. The cloning strategy has been previously described (Korea et al., 2014). Briefly, Δ*esxA and* Δ*esxB* single deleted mutants were generated by cloning the respective *esx* gene into the *Escherichia coli*-*S. aureus* shuttle/suicide vector pKOR1; while *esxA* gene was deleted from Δ*esxB* strain to obtain the Δ*esxAB* double mutant. All isogenic mutants were tested for the correct expression of *esxA* and *esxB* genes by PCR, using external primers targeting flanking regions, and by sequencing the genomic region corresponding to the deleted genes (*esxA* and *esxB*), including upstream and downstream regions (500–700 bp; Korea et al., 2014). Green Fluorescent Protein (GFP)-expressing bacterial strains were instead obtained by cloning pOS1CK-GFP vector into wt and *esx* mutants (Korea et al., 2014).

Fresh bacterial preparations were cultured for each experiment. Briefly, wt USA300 and *esx* mutants were grown in tryptic soy broth (Becton Dickinson, Sparks, MD, USA) overnight at 37°C. The next day, bacterial broths were diluted 1:100 in fresh tryptic soy broth, cultured until the exponential phase of growth (OD_600_ of 0.6), and then washed in RPMI 1640 and re-suspended in RPMI 1640 supplemented with L-glutamine (2 mM) and 15% FBS for DC infection.

### Colony forming units (CFU) count

DC were infected with wt USA300, Δ*esxAB*, Δ*esxA*, and Δ*esxB* at multiplicity of infection of 0.1. At the indicated time points, cells were harvested and washed in RPMI 1640 by centrifugation at 150 × g for 10 min to selectively spin down cells, while extracellular bacteria remained in the supernatants. Collected cells were lysed with distilled water containing 0.1% saponin for 5 min at room temperature and then plated at serial dilutions on tryptic soy agar (Becton Dickinson) plates to obtain the number of CFU/ml.

### Confocal microscopy

DC were infected for 4 h with wt USA300 and *esx* mutants expressing GFP. After infection, DC were harvested, washed and then fixed with 4% paraformaldehyde. Cells were subsequently washed with PBS and incubated with phalloidin-Tritc (Sigma Aldrich) for 1 h to highlight the actin filaments (red). Nucleic acids (blue) were labeled with far-red fluorescent dye RedDot™2 (Biotium, Fremont, CA, USA) following an incubation of 30 min in PBS solution. Vectashield Antifade Mounting Medium (Vector Laboratories, Burlingame, CA, USA) diluted at 80% in PBS was used to prepare specimens for confocal microscopy observation. Images were acquired with Leica TCS SP5 confocal microscope and processed with LAS AF software (version 1.6.3, Leica Microsystems). Objective 63.0X. Lasers activated: He/Ne laser at 543 nm to phalloidin-Tritc's excitation, He/Ne laser at 633 nm to dye RedDot™2's excitation and Argon laser at 488 nm to visualize GFP-*S. aureus* (green). Images were acquired activating single laser in sequential mode to prevent fluorescence overlay. Image magnification 1575X, pixels 1,024 × 1,024.

### Antibodies and other reagents

Monoclonal antibodies (Abs) specific for cluster of differentiation (CD)1a, CD14, CD38, CD86, CD83, HLA-DR, IgG1, IgG2a (BD Bioscience, San Diego, CA, USA), and Annexin V (Abcam, Cambridge, UK) were used as direct conjugates to Fluorescein Isothiocyanate (FITC), Phycoerythrin (PE) as needed. To exclude dead cells from analysis Fixable Viability Dye eFluor®780 (FvDye, eBioscience, San Diego, CA, USA) was used. For immunoblotting analysis rabbit anti-phospho-NF-kB p65 (Ser536), rabbit anti-phospho-p44/42 MAPK (Thr202/Tyr204), rabbit anti-phospho-p38 MAPK (Thr180/Tyr182) (Cell Signaling Technology, Danvers, MA), mouse anti-β-actin (Sigma-Aldrich, St. Louis, MO, USA) and horseradish peroxidase-conjugate anti-mouse (Santa Cruz Biotechnology, Dallas, TX, USA) and anti-rabbit (Santa Cruz Biotechnology) secondary Abs were used. Staurosporine (1 μM, Sigma Aldrich) was utilized as pharmacological inducer of apoptosis.

### Flow cytometry analysis

Cells (10^5^) were washed once in PBS containing 2% FBS and incubated with indicated Abs at 4°C for 30 min. DC were then washed and fixed with 2% paraformaldehyde (Panreac Quimica, Castellar del Valles, Spain) before analysis on Gallios cytometer (Beckman Coulter, Brea, CA, USA). A total of 30,000 events were analyzed per sample in FvDye negative live cells. The expression of cell surface molecules was evaluated in viable DC using the median fluorescence intensity (MFI) after subtraction of the respective isotype control Ab values.

### Apoptosis detection

Phosphatidylserine exposure and membrane integrity were analyzed by using Annexin V-FITC and FvDye according to manufacturing protocols. Twenty-four hours post infection, DC were stained with FvDye and then labeled for 15 min with 5X Annexin V-FITC, as previously described (Etna et al., 2014). Finally, cells were fixed overnight with 4% paraformaldehyde before analysis on Gallios cytometer (Beckman Coulter). Data were analyzed by Kaluza software (Beckman Coulter).

### Cytokine determination

Supernatants of DC cultures were harvested 24 h after infection, filtered with 0.2 μm devices and then stored at −80°C. The production of IL-12, TNF-α, IL-10, IL-1β, IL-6, and IL-8 was measured by human Inflammatory Cytokine kit (Cytometric Bead Array, BD Bioscience), while IL-23 release was quantified by ELISA (R&D Systems). Supernatants from stimulated CD4^+^ T cells were collected after 5 days, filtered and stored at −80°C. IFN-γ and IL-17 production was determined by specific ELISA kits (R&D Systems).

### Immunoblot analysis

Western blot were performed as previously described (Etna et al., 2015). Briefly, 25 μg of total protein extracts were separated on 10% SDS-PAGE gel and blotted onto nitrocellulose membranes (Millipore). Blots were incubated with rabbit polyclonal anti-phospho-p38 MAPK (Thr180/Tyr182) (p-p38), rabbit polyclonal anti-phospho-p44/42 MAPK (Thr202/Tyr204) (p-p44/42) and rabbit polyclonal anti-phospho-NF-kB p65 (Ser536) (p-p65) Abs (Cell Signaling Technology, Danvers, MA). Detection was achieved using anti-rabbit horseradish peroxidase-conjugate secondary Ab (Santa Cruz Biotechnology), and visualized with Enhanced Chemiluminescence plus kit (GE Healthcare Bio-Sciences, Pittsburgh, PA, USA). A ChemiDoc XRS (Bio-Rad, Hercules, CA, USA) instrument and ImageLab software (Bio-Rad) were used to reveal and analyze the chemiluminescence signal. For loading control, β-actin levels were quantified by using a mouse anti-β-actin Ab (Sigma Aldrich). Protein amount was normalized to the actin level by ImageLab software. Fold changes of each analyzed protein were calculated by dividing values obtained in infected conditions by those of the uninfected counterpart.

### T cell activation and expansion

Total CD4^+^ T cells were isolated from freshly collected buffy coat as previously described (Etna et al., 2014). Briefly, CD4^+^ T cells were purified by indirect magnetic sorting with a CD4^+^ T-cell isolation kit (Miltenyi, Teterow, Germany) from allogeneic PBMC. Purified cells were plated in 96-well U-bottomed tissue culture plates at the density of 0.4 × 10^6^ cells/ml with supernatants obtained from control or 24 h-infected DC. Conditioned T cells were stimulated with anti-CD3/CD28 beads (Invitrogen life technology, Carlsbad, CA, USA) (ratio 1:1) for 5 days and then supernatants were harvested for IFN-γ and IL-17 detection.

### Bioinformatics analysis

Prediction of the three-dimensional structure of EsxB has been performed by using *Rosetta* (http://robetta.bakerlab.org/queue.jsp) and I-TASSER (http://zhanglab.ccmb.med.umich.edu/I-TASSER/) software packages (Supplementary Figure 1). *Rosetta* parses the sequence into putative domains, looking for regions into the query sequence that are homologous to experimentally determined structures. Then proceeds with multiple sequence alignment (MSA) to predict putative domains. If a confident match to a protein of known structure is found, that is used as a template for comparative modeling. If no match is found, structure predictions are made using the *de novo* Rosetta fragment insertion method (Kaufmann et al., 2010). Once the protein chain is completely assembled, the side-chains of the final model are repacked using a Monte Carlo algorithm with a backbone-dependent sidechain rotamers library (Kaufmann et al., 2010). I-TASSER identifies template proteins, which are predicted to display a fold similar to that of the protein of interest using several threading techniques implemented in LOMETS. Structure fragments excised from the templates are then assembled through replica-exchange Monte Carlo simulations. Finally, the models obtained are iteratively refined to optimize their free energy and global topology (Roy et al., 2010).

*Dali server* has been used to detect structural homologs of EsxA and EsxB in the Protein Data Bank (see Supplementary Materials). The program has retrieved a list of 500 structural neighbors, ranked by highest Z-score, and the alignment data (Holm and Rosenstrom, 2010). Among all structural homologs, only human proteins have been further analyzed.

Human interactors of the proteins selected by the Dali server have been identified using the PrePPI server (http://bhapp.c2b2.columbia.edu/PrePPI). PrePPI combines the prediction of protein-protein interactions (PPI) with those coming from experimental evidence (Zhang et al., 2013). Predicted interactions are derived from calculated likelihood ratios (LRs) by combining structural, functional, evolutionary and expression information, with the most important contribution coming from structure. The experimentally determined interactions are collected from six publicly available databases (MIPS, DIP, IntAct, MINT, HPRD, and BioGRID) that manually collect PPIs from the literature and are also assigned LRs. A final probability is then assigned to every interaction by combining the LRs for both predicted and experimentally determined interactions (Zhang et al., 2013).

cNLS Mapper (http://nls-mapper.iab.keio.ac.jp/cgi-bin/NLS_Mapper_form.cgi) has been used for the prediction of the classical importin-alpha/beta nuclear localization sequence (NLS) in EsxA and EsxB amino acid sequences. cNLS mapper calculates NLS activity scores by using activity-based, but not sequence-based, profiles for different classes of importin-α-dependent NLSs. Higher scores indicate stronger NLS activities. Score values between 8 and 10 indicate that the protein is exclusively localized in the nucleus, score values of 7 or 8 that it is partially localized in the nucleus, score values between 3 and 5 that it is localized both in the nucleus and in the cytoplasm, and score values between 1 and 2 that it is localized exclusively to the cytoplasm (Kosugi et al., 2009).

Protein-Protein docking simulations to evaluate the feasibility of formation of EsxA-EsxB heterodimers and heterotetramers were performed using the ZDOCK online server (http://zdock.umassmed.edu, version 3.0.2), which is based on the rigid-body protein-protein docking program ZDOCK (Pierce et al., 2014). ZDOCK implements a Fast Fourier Transform algorithm and a scoring system based on a combination of shape complementarity, electrostatics and statistical potential terms (Pierce et al., 2014). The 2,000 complexes predicted by ZDOCK were re-ranked using ZRank (Pierce and Weng, 2007), a program that rescores the ZDOCK predictions using a more detailed potential which includes electrostatics, van der Waals, and desolvation terms (Pierce and Weng, 2007). Results of the above described bioinformatics analyses are reported in the Supplementary Material.

### Statistical analysis

Statistical analysis was carried-out using a two-tailed Student's *t*-test for paired data. A *P* < 0.05 was considered statistically significant.

## Results

### Infection of human DC with wt USA300 and *esx* deletion mutants

In light of the importance of DC in the surveillance of peripheral sites and in the induction of an effective immune response, we sought to explore the role of *S. aureus* EsxA and EsxB proteins by infecting DC with the isogenic Δ*esxA* and Δ*esxB* single mutants as well as the Δ*esxAB* double mutant generated in *S. aureus* USA300 background (Korea et al., 2014).

**Figure 1:**
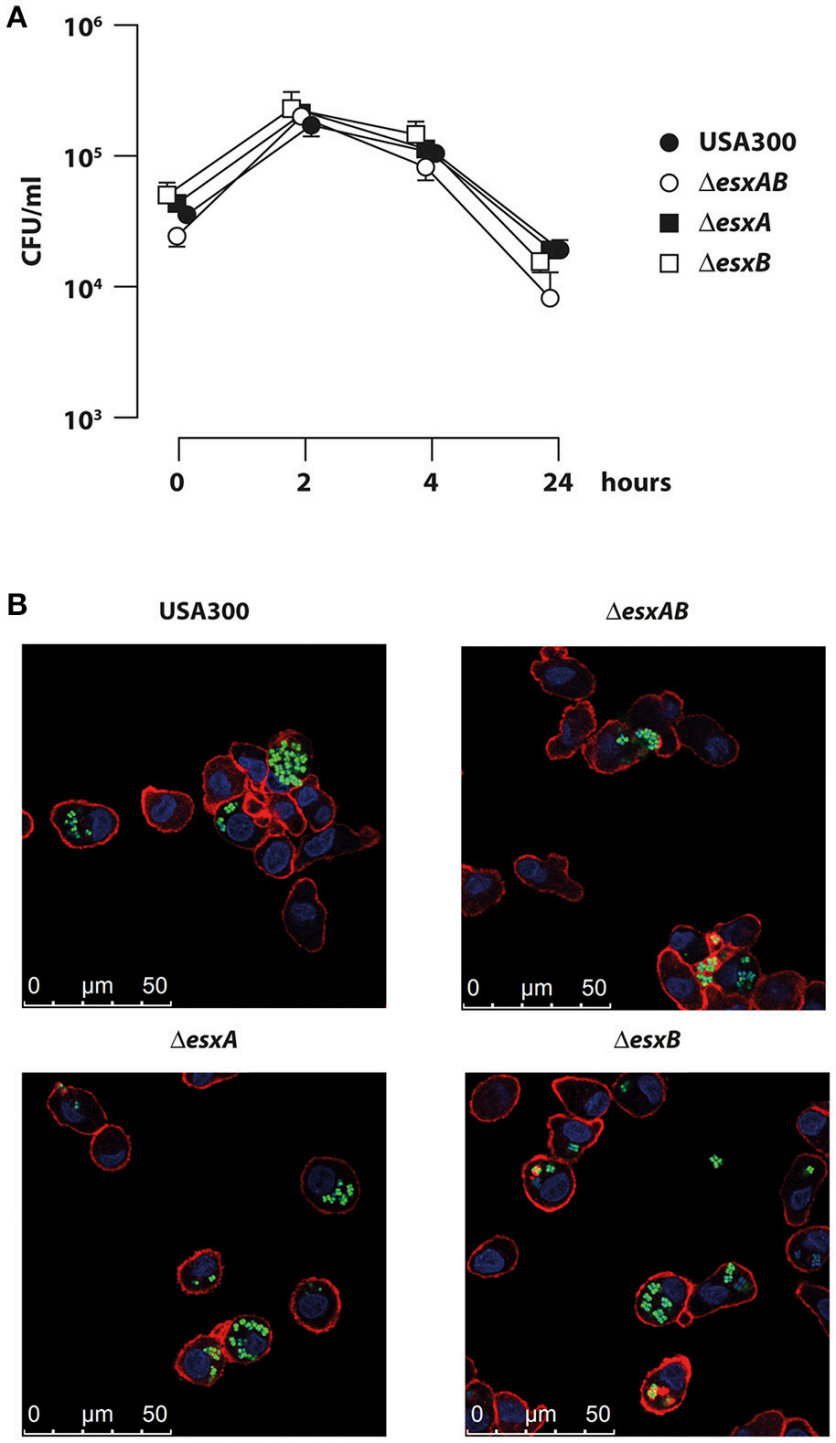
Infection of human DC with wild type USA300 and *esx* mutants. DC were infected with wild type USA300 or Δ*esxAB*, Δ*esxA*, and Δ*esxB* mutant strains for the indicated time points. **(A)** Cells were extensively washed to eliminate extracellular bacteria and then lysed for intracellular bacteria quantification by CFU counting. The values are the mean ± SEM of 3 independent experiments. **(B)** DC were infected with wild type USA300 or *esx* mutants expressing GFP. Four hours after infection cells were analyzed for bacteria internalization by confocal microscopy following DC cell surface and nucleus visualization using phalloidin-TRITC probe and DAPI, respectively. Scale bar 0–50 μm.

In agreement with a previous report demonstrating the presence of *S. aureus* inside DC or on their cell surface (Jin et al., 2014), we observed that human DC actively phagocytized *S. aureus* (Figure 1). In particular, the comparative analysis of the intracellular CFU number showed that DC efficiently internalized wt USA300 as well as the isogenic *esx* mutants (Figure 1A). A time course analysis showed that CFU counts within DC increased up to 2 h post infection and then gradually decreased during the following 22 h. Importantly, CFU counts showed that the number of the internalized wt USA300 and mutant strains was similar at all analyzed time points, suggesting that EsxA and EsxB do not directly affect the ability of *S. aureus* to infect DC cultures. Confocal microscopy experiments performed with GFP-expressing wt USA300 and *esx* recombinants, confirmed CFU data showing no differences among the internalized bacteria (Figure 1B).

### Induction of DC maturation and apoptosis in response to the infection with wt USA300 and *esx* deletion mutants

**Figure 2:**
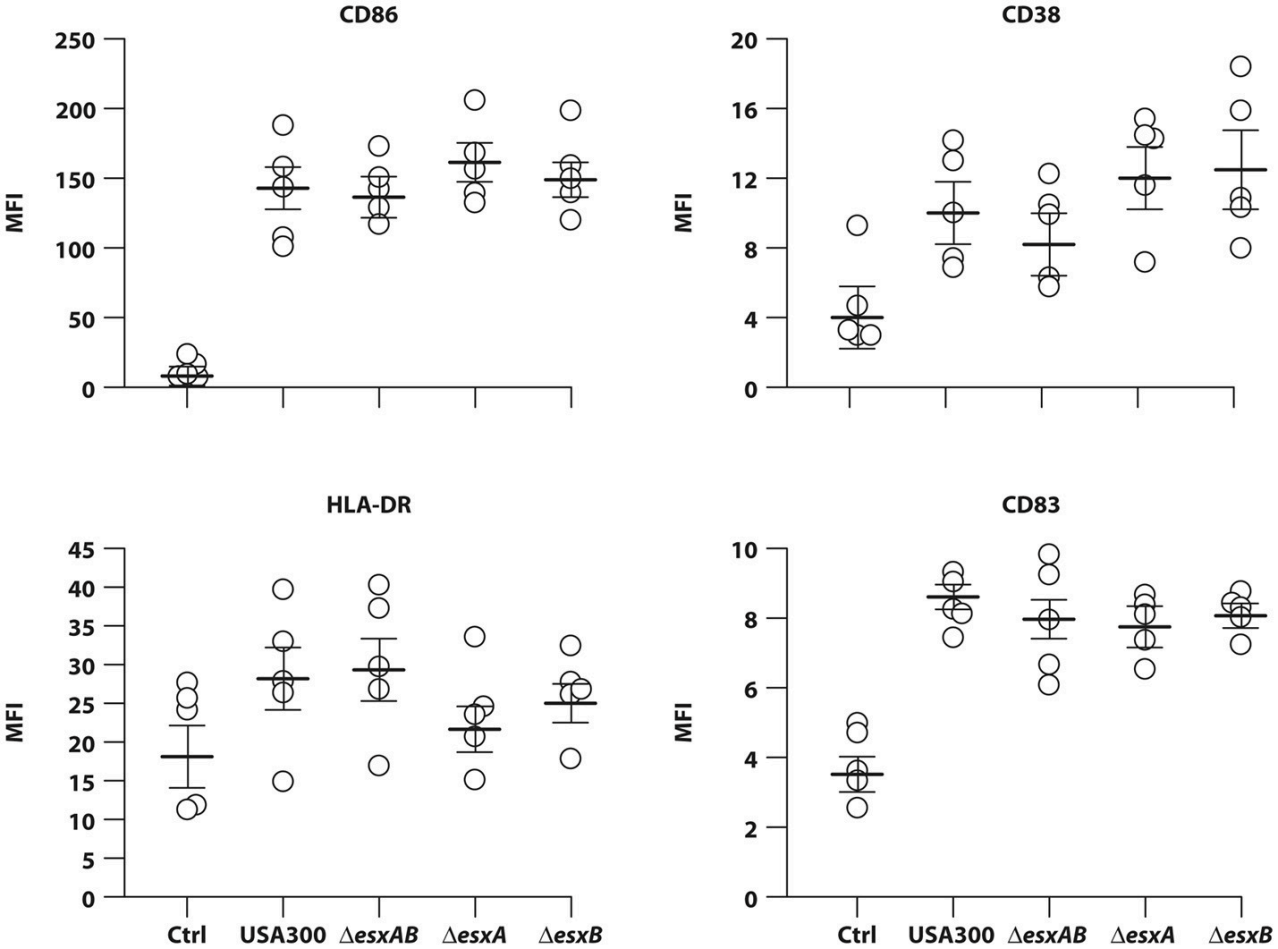
DC maturation in response to infection with wild type USA300 or Δ*esxAB*, Δ*esxA*, and Δ*esxB* mutants. DC were left untreated (Ctrl) or infected for 24 h with wild type USA300 or with USA300 mutants deleted in the *esx* factors. Surface expression of CD86, CD38, HLA-DR, and CD83 was examined by cytometric analysis in 5 independent experiments and graphed by calculating the mean fluorescence intensity (MFI) after the subtraction of the isotype Ab controls. MFI values for each individual experiment and mean MFI ± SEM are shown.

Having found that wt USA300 and *esx* deletion mutants displayed a similar ability to infect human DC cultures, next we evaluated how the infection impacts on DC immune-phenotype. In particular, DC were infected for 24 h with wt USA300, Δ*esxAB*, Δ*esxA*, and Δ*esxB* mutants and, then, the surface expression of the co-stimulatory molecule CD86, the maturation marker CD83, the MHC class II molecule HLA-DR and the activation marker CD38 was examined by flow cytometry. All strains induced a robust expression of the analyzed molecules as compared to the uninfected counterpart irrespective of the presence of Esx-encoded factors (Figure 2), suggesting that Esx proteins do not interfere with this process.

Given the importance of the apoptosis in antigen presentation and, consequently, in the commitment of T cell response, we analyzed the percentage of Annexin-V and FvDye labeled DC 24 h after infection with wt USA300 or Δ*esxAB*, Δ*esxA*, and Δ*esxB* mutants.

**Figure 3:**
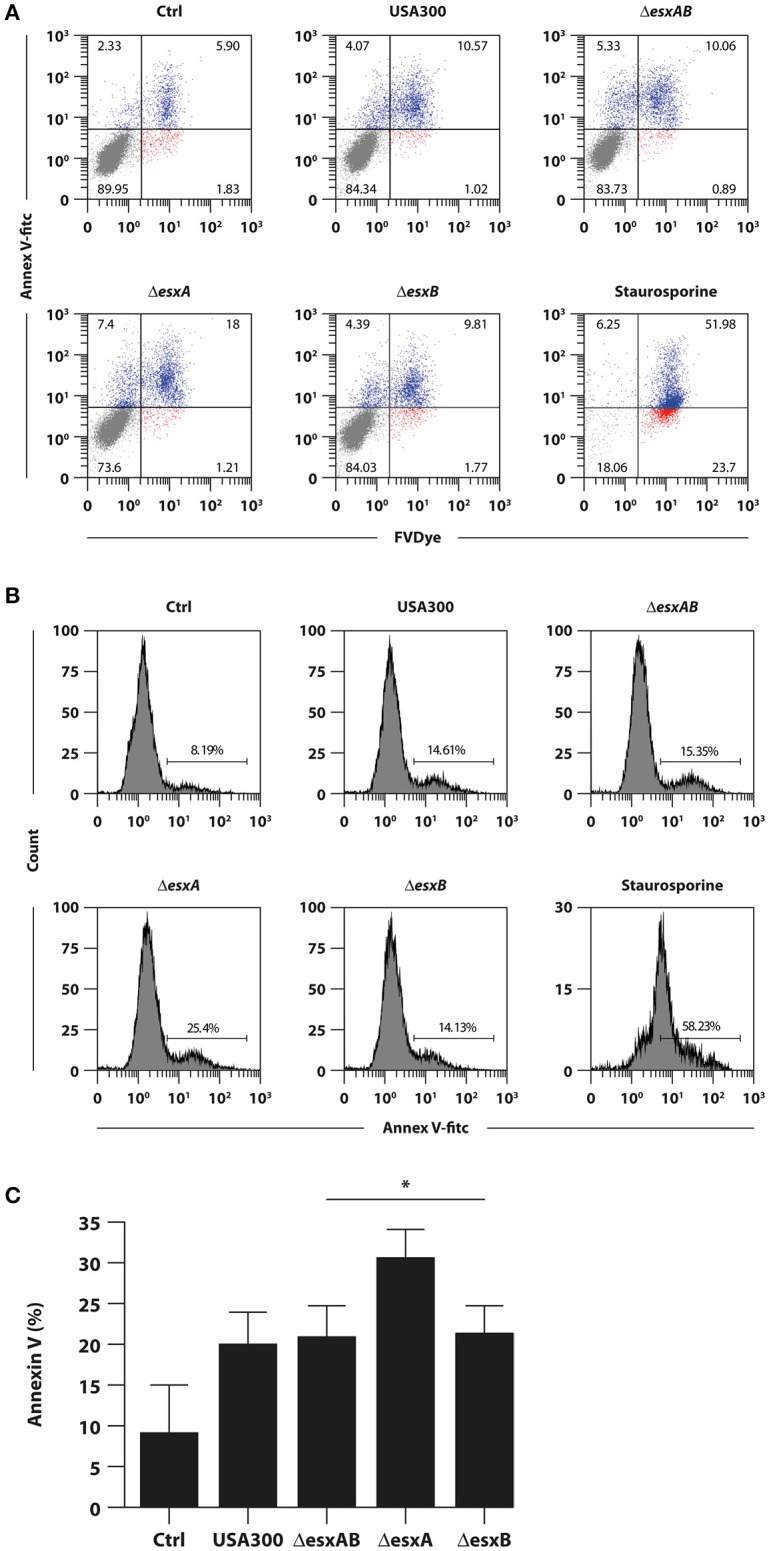
Impact of wild type USA300 and *esx* mutants on DC apoptosis. DC were left untreated (Ctrl) or infected for 24 h with wild type USA300 or Δ*esxAB*, Δ*esxA*, and Δ*esxB* recombinants. **(A)** Apoptosis was assessed by cytofluorimetric analysis through Annexin-V and FvDye staining. As internal control DC were treated with 1 μM staurosporine for 16 h. A representative experiment, out of 3 independent experiments performed that yielded similar results, is shown. Numbers in the dot plots correspond to the percentage of cells in each quadrant. **(B)** The expression of Annexin-V for the different experimental conditions was reported as the histogram of fluorescence intensity. Numbers in each panel correspond to the percentage of total Annexin-V positive cells. **(C)** Histogram of the means of Annexin-V positive cell percentage ± SEM of 3 independent experiments. Statistical significance was calculated by Student's *t*-test for paired data. ^*^P = 0.004 (wild type USA300- vs. ΔesxA recombinant-infected DC). Statistics refers to strains at the extremity of the horizontal lines.

Infection with all analyzed strains increased the percentage of both early (annexin^+^/FvDye^−^) and late (annexin^+^/FvDye^+^) apoptotic cells as compared to the uninfected counterpart (Figure 3A). However, Δ*esxA* mutant infected DC displayed a significantly stronger apoptotic phenotype respect to wt USA300 infected cultures (Figures 3B,C).

### Cytokine production by DC infected with wt USA300 and *esx* deletion mutants

**Figure 4:**
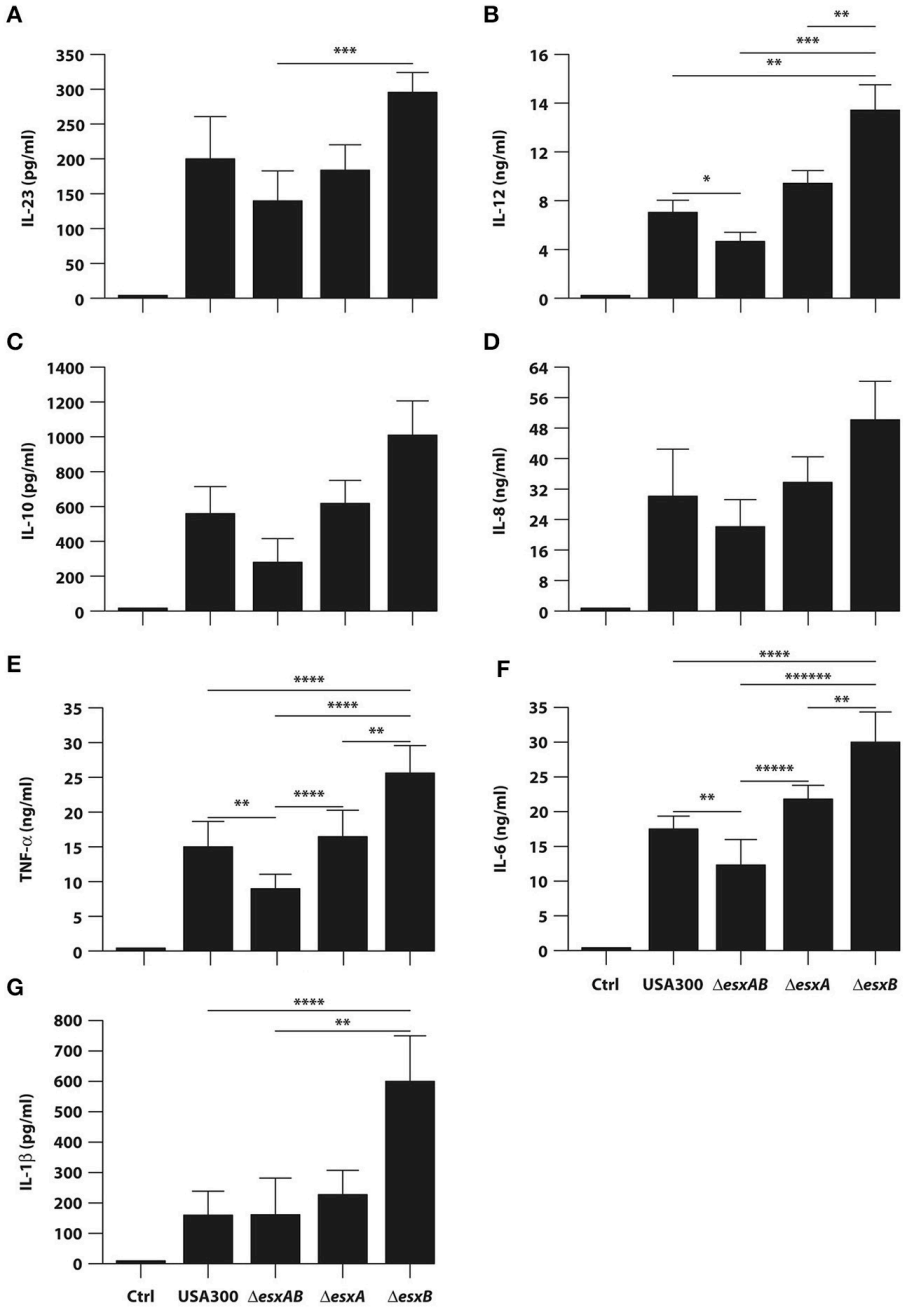
Cytokine production by DC infected with wild type USA300 or *esx* mutants. DC were left untreated (Ctrl) or infected for 24 h with wild type USA300 or Δ*esxAB*, Δ*esxA*, and Δ*esxB* mutants. **(A–G)** IL-23 production was measured in DC culture supernatants by ELISA while, the secretion of IL-12, IL-10, IL-8, TNF-α, IL-6, and IL-1β was instead evaluated by Inflammatory Cytokine array kit. The results represent means ± SEM of 6 independent experiments. Statistical Significance was calculated by Student's *t*-test for paired data. ^*^P = 0.003; ^**^P < 0.05; ^***^P < 0.01; ^****^P < 0.02; ^*****^P = 0.03; ^******^P = 0.006. Statistics refers to strains at the extremity of the horizontal lines.

In an attempt to evaluate whether the presence or absence of Esx proteins in USA300 bacteria would affect the ability of DC to secrete soluble immune mediators, DC culture supernatants were collected 24 h after infection with wt USA300 or the *esx* isogenic mutants, and the cytokine production was analyzed. Both wt USA300 and all isogenic mutants drove production of regulatory cytokines (IL-23, IL-12, and IL-10) and pro-inflammatory mediators (TNF-α, IL-6, and IL-1β), as well as the chemokine IL-8, although at different extent (Figure 4). In particular, the single *esxB* deletion conferred to USA300 an increased capacity to promote the release of IL-12, TNF- α, IL-6, and IL-1β (Figures 4B,E–G).

**Figure 5:**
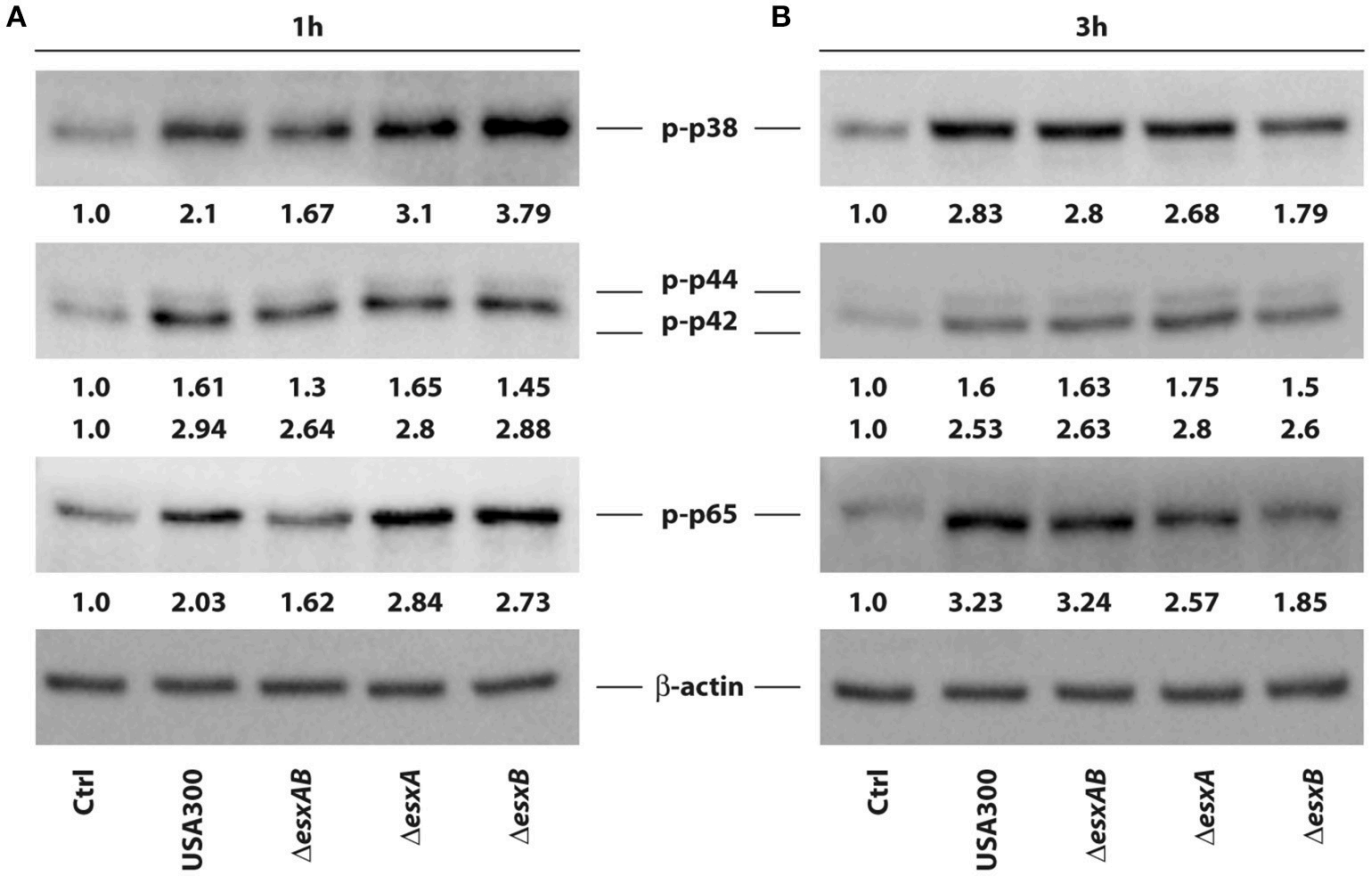
Activation of p38, p44/42 MAPK and NF-kB p65 in DC infected with wild type USA300 or *esx* mutants. The activation of intracellular pathway involving p38, p42/44 MAPK, and NF-kB p65 subunit was investigated by western blotting analysis of the phosphorylated isoforms in whole cell extracts prepared from DC infected for 1 **(A)** and 3 h **(B)** with wild type USA300 or *esx* mutants. β-actin levels were analyzed as control for protein loading. Fold changes of each analyzed protein, indicated at the bottom of each immunoblot, were calculated by dividing values obtained in infected conditions with those of the uninfected counterpart. A representative experiment, out of 3 independent experiments performed that yielded similar results, is shown.

Given the differences observed in cytokine production, we investigated the signaling pathways involved in the transcriptional regulation of these factors in response to the infection by wt USA300 and *esx* deletion mutants. In particular, MAPK and NF-kB activation was explored in DC challenged with either wt USA300 or *esx* mutants. In accordance with the observed modulation of DC cytokine milieu, phosphorylation of the MAPK p38 was induced 1 h after infection with both wt USA300 and mutants, although to a different extent. Indeed, while Δ*esxAB* recombinant poorly enhanced p38 phosphorylation, Δ*esxB* mutant resulted to be the strongest inducer as compared to wt USA300 and the other recombinant strains (Figure 5A). The p38 activation profile correlates with the down-stream phosphorylation of the NF-kB p65 subunit. Indeed, while Δ*esxAB* mutant weakly stimulated p65 activation, both Δ*esxA* and Δ*esxB* recombinants induced a robust phosphorylation of this NF-kB subunit (Figure 5A).

At 3 h post-infection both p38 and p65 activation was lowered in DC infected with the Δ*esxB* strain as compared to the other mutants, who displayed a sustained phosphorylation of both molecules (Figure 5B).

Finally, no difference was found in p42/44 (ERK1/2) phosphorylation 1 and 3 h after the infection with all analyzed *S. aureus* strains, although a slight increase of p42 phosphorylation was observed in Δ*esxA*-infected DC (Figure 5).

Despite the observed differences are not statistically significant, wt USA300 and *esx* mutants may stimulate intracellular signaling activation and, consequently, cytokine production in DC with different kinetics and to a different extent.

### Influence of wt USA300 and *esx* mutant-induced cytokine milieu on IFN-γ and IL-17 release from CD4^+^ T cells

Finally, to investigate the impact of cytokine milieu released by infected DC on the expansion of IFN-γ and IL-17 producing T cells, we stimulated CD4^+^ T cells with anti-CD3/CD28 beads in presence of supernatants obtained from heterologous DC cultures infected for 24 h with either wt USA300 or Δ*esxAB*, Δ*esxA*, and Δ*esxB* mutants.

**Figure 6:**
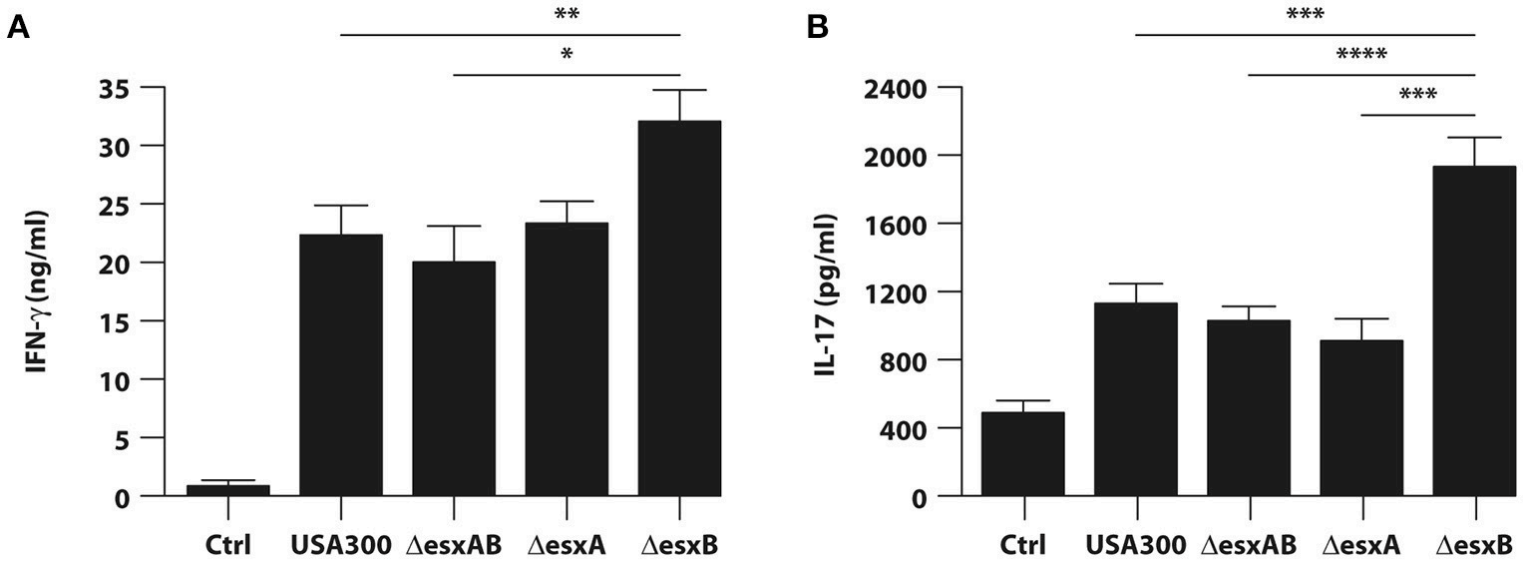
Expansion of IFN-γ or IL-17 producing T cells by cytokine milieu released from DC infected with wild type USA300 and *esx* mutants. Total CD4^+^ T cells were stimulated with anti-CD3/CD28 magnetic beads in presence of supernatants derived from DC cultures untreated or infected for 24 h with wild type USA300 or *esx* mutants. IFN-γ **(A)** and IL-17 **(B)** production was evaluated in harvested supernatants by ELISA. The results shown represent means ± SEM of 4 independent experiments. Significance was calculated by Student's *t*-test. ^*^P = 0.006; ^**^P = 0.01; ^***^P = 0.02; ^****^P = 0.05.

In accordance with the high level of IL-12 produced in response to both wt USA300 and *esx* mutant infection (see Figure 4B), IFN-γ was strongly released by T cells in all the analyzed experimental conditions (Figure 6A). Furthermore, IFN-γ secretion was significantly higher when T cells were stimulated with supernatants derived from Δ*esxB*-infected DC than what induced by the other supernatants (Figure 6A). A similar trend was observed for Th17 expansion (Figure 6B), since the highest IL-17 production was observed in CD4^+^ T cell culture conditioned by supernatants of Δ*esxB*-infected DC.

Collectively, these data indicate that *esxB* deletion confers to USA300 a stronger capacity to promote in DC a cytokine production optimal for the expansion of potentially protective Th1/Th17 response as compared to both wt and the other deletion mutants.

## Discussion

The specialized type VII secretion system found in Gram-positive *Actinobacteria* and *Firmicutes*, plays a variety of roles in bacterial physiology and pathogenesis (Green and Mecsas, 2016). The effector molecules transported by this secretion system are often involved in a wide range of important functions, such as survival in different environment, manipulation of the host response and establishment of a replicative niche (Groschel et al., 2016; Bottai et al., 2017).

The first identified and the best characterized type VII machinery is the mycobacterial ESX-1 system (Bottai et al., 2017). In the context of *M. tuberculosis* infection, several cellular events have been demonstrated to be manipulated by the activity of a functional ESX-1 system: inhibition of phagosomal maturation and acidification (Deretic et al., 2009), cytosolic access, autophagy block (Romagnoli et al., 2012), host cell-death (Derrick and Morris, 2007), modulation of the inflammatory (Stanley et al., 2003), and adaptive immune response (Etna et al., 2015), indicating that this secretion apparatus constitutes a major virulence factor (Green and Mecsas, 2016). Less defined is the role played by the *S. aureus* Ess in the modulation of host immune response. Notwithstanding the type VII-like Ess of *S. aureus* and the mycobacterial ESX-1 system are phylogenetically distant, these systems share two types of conserved elements: the membrane-bound ATPase of the FtsK/SpoIIIE that forms hexameric membrane-spanning pores, and the secreted WXG100 proteins (Jager et al., 2016).

Based on this evidence and having found that mycobacterial ESX-1 system profoundly impacts on human DC functions (Romagnoli et al., 2012; Etna et al., 2015), here we sought to investigate how and whether *S. aureus* Ess is able to play a similar role.

In the context of *S. aureus*-DC interplay, some analogies were observed with the mycobacterial counterpart with respect to the ability to modulate the apoptotic process (Derrick and Morris, 2007; Choi et al., 2010; Aguilo et al., 2013; Etna et al., 2015). Indeed, the *S. aureus* mutant lacking the expression of EsxA factor significantly promotes apoptosis in DC with respect to wt USA300 strain (Figure 3) in agreement to previous data by Korea and collaborators indicating an inhibitory role of EsxA protein on the apoptotic process in human epithelial cells (Korea et al., 2014). Nevertheless, unexpected data were obtained when Δ*esxAB* double mutant was used to infect DC since the apoptotic process was not impaired but even induced at a level similar to that present in wt USA300-infected DC (Figure 3) likely suggesting a not yet characterized role of EsxB factor in controlling the apoptotic process. This result was also in contrast with the increased apoptosis observed by Korea and collaborators in Δ*esxAB* mutant-infected epithelial cells (Korea et al., 2014). A possible explanation for this opposing data may rely on the different cell type in which the apoptotic process was analyzed, suggesting that the Esx factors could play a different role depending on the infected cell.

Another important finding of our study relies on the differential expression of regulatory and inflammatory cytokines (Figure 4), in spite of the absence of significant differences in the induction of DC maturation in response to the infection with wt USA300 and *esx* mutant strains (Figure 2). Indeed, all strains induced a robust expression of the analyzed maturation markers as compared to the uninfected counterpart irrespective of the presence of Esx-encoded factors suggesting that Esx proteins do not interfere with this process (Figure 2). Instead, our comparative analysis of cytokine production revealed that the induction of both regulatory (IL-12) and pro-inflammatory (TNF-α, IL-6, IL-1β) cytokines was higher in DC infected with the isogenic mutant lacking *esxB* expression respect to wt USA300 or the other recombinant strains (Figure 4). Surprisingly, this cytokine induction was reduced in DC cultures infected with Δ*esxAB* mutant (Figure 4). This unexpected and apparently counterintuitive result may be explained by the reported capacity of *S. aureus* to express other Esx factors (Anderson et al., 2013). In particular, Anderson and collaborators have described that, although the deletion of either *esxA* or *esxB* blocks the secretion of all Esx factors in USA300, different effects on Esx production were observed: the absence of *esxA* does not affect the expression of EsxB, EsxC, and EsxD while the deletion of *esxB* impairs only the production of EsxD (Anderson et al., 2013).

Moving from Anderson's data, we could speculate that Esx proteins could be released by dying bacteria and, once in the cell cytosol, they might interact with host factors/sensors. Thus, the different combination of multimeric complexes or single Esx subunits, which are present in knockout mutants, might contribute to the DC phenotype observed in this manuscript. Therefore, by tuning Esx balance, *S. aureus* could modulate the host cytokine production and programmed cell death (Fraunholz and Sinha, 2012), complex events at the crossroad of host's defensive responses and the pathogen's virulence and evasion mechanisms. This is an intriguing hypothesis whose demonstration requires additional analysis, including the complementation of the deleted target genes to formally demonstrate the specific role of EsxA and EsxB factors in controlling DC immune-regulatory functions.

Interestingly, our data on Esx factor-mediated modulation of cytokine expression are in line with findings from Anderson and collaborators, who demonstrated that, in a mouse model of *S. aureus* bloodstream infection, the EssE-mediated secretion of effectors proteins via the Ess pathway contributes to the manipulation of host immune response by affecting the production or the suppression of specific cytokines (Anderson et al., 2016). These findings are also important in light of the effect of cytokine production on anti-staphylococcal immunity. In this context, it is conceivable that the enhanced release of IL-12, TNF-α, IL-1β, and IL-6 by Δ*esxB* infection likely promotes a robust Th1 and Th17 response (Figure 6), whose crucial role in the immunity against *S. aureus* has been demonstrated by several studies (Spellberg et al., 2008; Lin et al., 2009; Krishna and Miller, 2012; Zielinski et al., 2012; Bagnoli et al., 2015; Kolata et al., 2015). In addition, in spite of several differences in the experimental setting, such as infection with live bacterium in our human DC-based model vs. the immunization with the recombinant staphylococcal proteins of the murine model, our data well correlate with the results by Zhang and collaborators showing that in mice EsxA and EsxB recombinant proteins promote both Th1 and Th17 responses (Zhang et al., 2015).

We also investigated the mechanisms underlying the cytokine production by studying kinetics of activation of MAPK p38 and NF-kB p65 pathways (Figure 5), known regulators of *S. aureus*-induced immune response (Krishna and Miller, 2012; Armbruster et al., 2016). We observed that wt USA300 and *esx* mutants stimulate intracellular MAPK and NF-kB activation with different kinetics and to a different extent. Although, the observed differences in MAPK and NF-kB activation were not statistically significant, these results suggest that *S. aureus esx* mutants might differentially control intracellular signaling involved in the regulation of cytokine expression.

In this regard, structural bioinformatics analysis revealed that both EsxA and EsxB factors share significant structural homology with the human proteins inhibitor growth factor 4 (ING4) and charged multivesicular body protein 4B (CHMP4B). Interestingly, ING4 is a factor involved in the regulation of NF-kB and p53 activity, which mediate different cellular functions including apoptotic process and cytokine production (Coles et al., 2010; Mathema and Koh, 2012) supporting our findings about the involvement of EsxA and EsxB in the modulation of DC apoptosis and cytokine secretion. Moreover, EsxA and EsxB homology with CHMP4B, a component of ESCRT-III (endosomal sorting complex required for transport III) required for degradation of surface receptor proteins and formation of endocytic multivesicular bodies (Ariumi et al., 2011), suggests a possible interference with the endocytic pathway, which could favor *S. aureus* escape from phagocytes. This hypothesis well correlates with data from Korea and collaborators demonstrating that both EsxA and EsxB are required for the release of *S. aureus* from the infected epithelial cells (Korea et al., 2014). In line with this view, it has been recently demonstrated that also the mycobacterial EsxH factor, secreted by the ESX-3 type VII secretion system, is able either to impair phagosome maturation or to undermine MHC class II presentation by disrupting ESCRT function (Mehra et al., 2013; Portal-Celhay et al., 2016) thus, supporting the idea on the interfering role of Esx factors with host response.

Although, our bioinformatics analysis predicts that EsxA and EsxB may mimic or interact with host-specific proteins, the mechanism by which EsxA and/or EsxB exert their function on DC immune response needs to be further investigated and experimentally validated in future study.

In the attempt to investigate EsxA and EsxB heterodimeric and/or multimeric protein complex formation, *in silico* bioinformatics analysis was performed indicating that both the heterodimeric and the heterotetrameric assembly of EsxA with EsxB is stereochemically feasible (Supplementary Figures 1, 2). The physical interaction between EsxA and EsxB factors has been hypothesized since their discovery (Burts et al., 2005), mainly on the basis of the similarity with the related mycobacterial proteins, although empirical demonstration of such a complex has not yet been obtained (Sundaramoorthy et al., 2008; Anderson et al., 2013). The experimental validation of the predicted EsxA-EsxB heterodimer/heterotetramer and the definition of the features of this interaction might help to explain the different behavior of the double mutant Δ*esxAB* with respect to both the single Δ*esxA* and Δ*esxB* mutants in the modulation of DC immune functions.

In conclusion, our findings indicate that staphylococcal Esx factors may influence DC functions and, in turn, the adaptive immune response fate providing important information, which could be exploited for developing innovative preventive or therapeutic interventions against *S. aureus*.

## Author contributions

MC: Conducted DC infection, sample preparation for confocal microscopy and FACS analysis, protein extraction, western blotting, T cell polarization experiments, predictive analysis of *S. aureus* Esx factor interaction, analyzed the results and wrote the manuscript; ME: Conducted sample preparation for FACS analysis, T cell polarization experiments, predictive analysis of *S. aureus* Esx factor interaction, analyzed the results and wrote the manuscript; RC and SS: Conducted *S. aureus* strain preparations and CFU counting; EG: Conducted DC preparation, analyzed the results and discussed the data; MS: Conducted FACS experiments, analyzed the results and discussed the data; FR: Conducted FACS experiments and analyzed the results; ZP: Conducted confocal microscopy experiments and analyzed the results; VB: Conducted predictive modeling and interaction analysis; FP: Conducted predictive modeling, interaction analysis and discussed the data; EA and AP: Analysis and discussion of the results; FB: Analysis, interpretation, discussion of the results and revised the manuscript critically; EC: Guided the idea generation, experimental work, and manuscript preparation. All authors reviewed the manuscript.

### Conflict of interest statement

AP has received an unrestricted grant from Merck Sharp and Dohme. FB is an employee of GSK Vaccines and owns patents on *S. aureus* vaccine candidates as well as GSK stocks. The author has no other relevant affiliations or financial involvement with any organization or entity with a financial interest in or financial conflict with the subject matter or materials discussed in the manuscript apart from those disclosed. The other authors declare that the research was conducted in the absence of any commercial or financial relationships that could be construed as a potential conflict of interest.

## Abbreviations

Abs: Antibodies
CHMP4B: Charged multivesicular body protein 4B
CD: cluster of differentiation
CFU: colony forming unit
DC: Dendritic cells
ESCRT: endosomal sorting complex required for the transport
ESCRT-III: endosomal sorting complex required for the transport complex III
Ess: ESAT-6-like secretion system
FBS: Fetal Bovine Serum
FvDye: Fixable Viability Dye
GFP: Green Fluorescent Protein
h: hours
ING4: Inhibitor Growth factor 4
LRs: likelihood ratios
MFI: mean fluorescence intensity
min: minutes
MSA: multiple sequence alignment
NLS: nuclear localization sequence
SEM: standard error of the mean
*S. aureus*: *Staphylococcus aureus*
wt: wild type.
