# Supplementary figures and images for: Staphylococcus aureus Esx Factors Control Human Dendritic Cell Functions Conditioning Th1/Th17 Response
Source: Front Cell Infect Microbiol. 2017 Jul 21;7:330. doi: 10.3389/fcimb.2017.00330 (PMC5519619; doi:10.3389/fcimb.2017.00330)

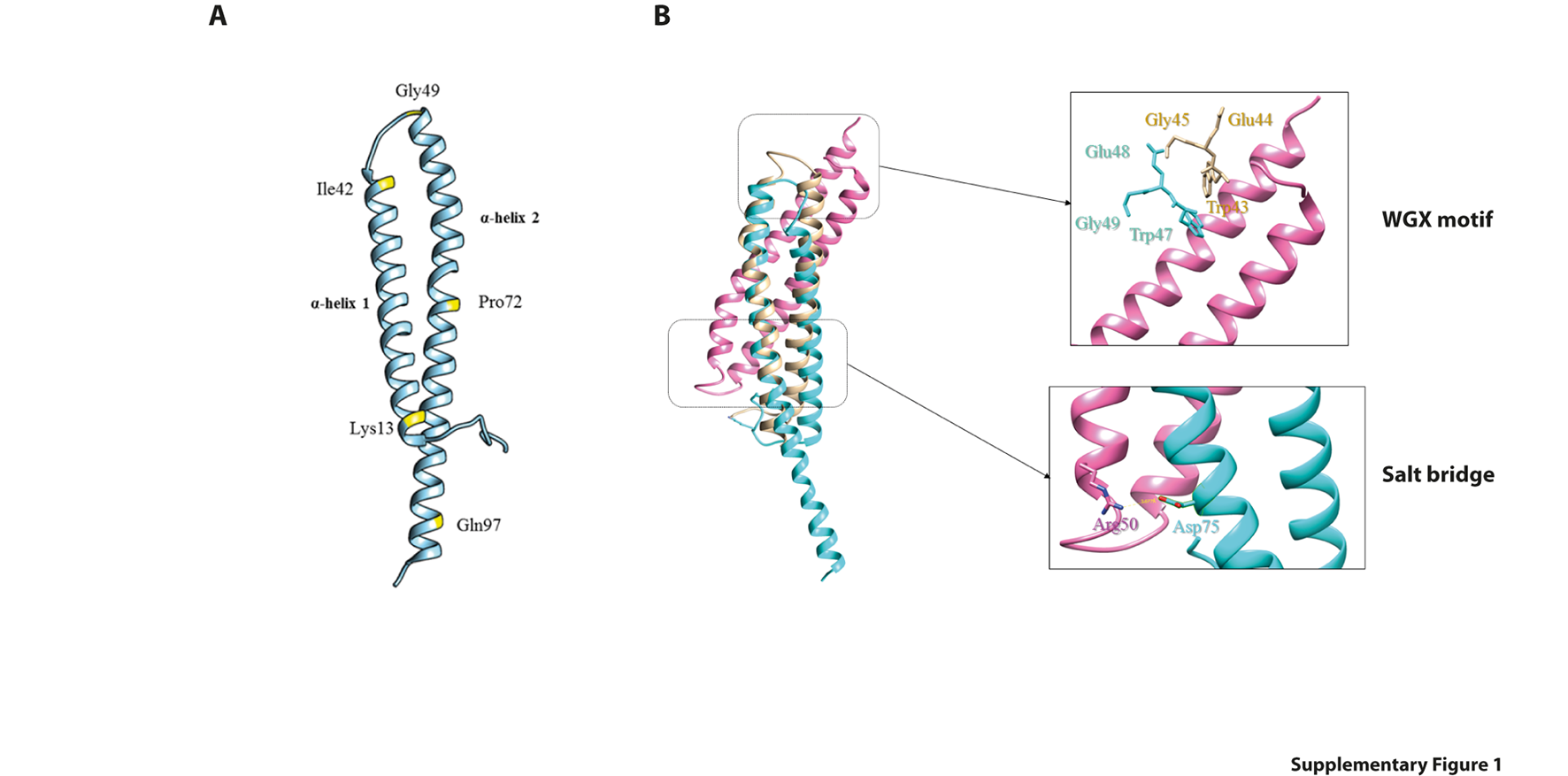

Supplement: Supplementary file 1 [file Image1.TIF]

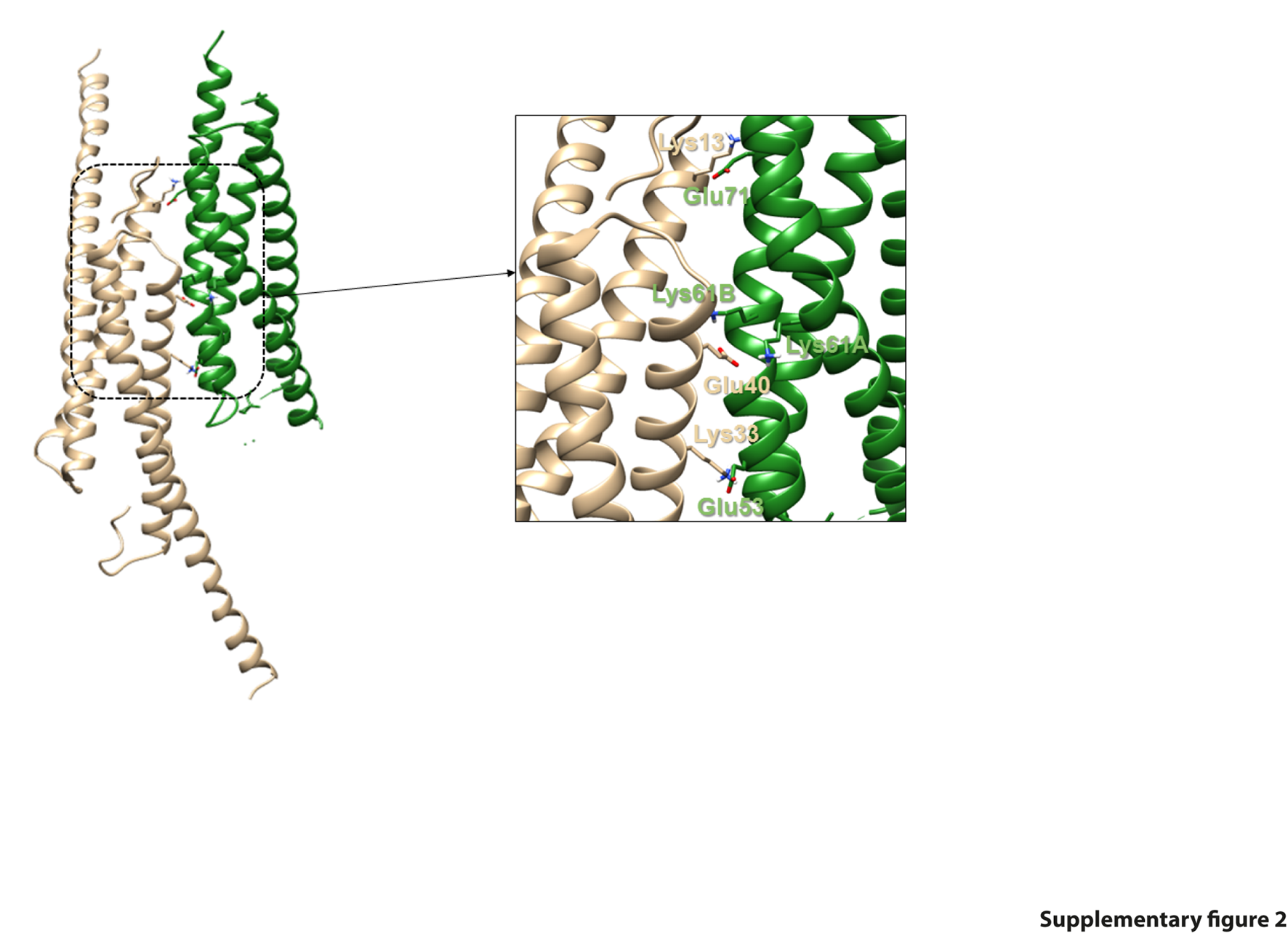

Supplement: Supplementary file 2 [file Image2.TIF]
